# Risk of fragility fracture is aggravated during bone regeneration processes in osteoporotic sheep

**DOI:** 10.1371/journal.pone.0319910

**Published:** 2025-05-02

**Authors:** Juan J. Toscano-Angulo, Juan Mora-Macías, Pablo Blázquez-Carmona, Juan Morgaz, Rocío Navarrete-Calvo, Jaime Domínguez, Esther Reina-Romo

**Affiliations:** 1 Department of Mechanical and Manufacturing Engineering, Escuela Técnica Superior de Ingeniería, Universidad de Sevilla, Sevilla, Spain; 2 Department of Mining, Mechanical, Energy and Building Engineering, Escuela Técnica Superior de Ingeniería, Universidad de Huelva, Huelva, Spain; 3 Department of Mechanical Engineering and Industrial Design, Escuela Superior de Ingeniería, Universidad de Cádiz, Puerto Real, Spain; 4 Department of Animal Medicine and Surgery, Facultad de Veterinaria, Universidad de Córdoba, Córdoba, Spain; University of Gothenburg: Goteborgs Universitet, SWEDEN

## Abstract

**Introduction:**

Bone regeneration processes are associated with a systemic skeletal change in bone quality, increasing the risk of fragility fractures. This condition may be aggravated in osteoporotic patients due to their limited osteogenic capacity. This work evaluates the impairment of the bone quality in osteoporotic sheep during a bone regeneration process. It provides a deeper understanding about the complex multiscale dynamics of bone mineral density, microstructure and chemical composition across different bone tissues, locations and time points.

**Materials and Methods:**

Osteoporosis was induced in fifteen Merino sheep. A critical-size defect was then created in the sheep’s right hind metatarsus and subsequently regenerated using distraction osteogenesis. The animals were randomly sacrificed during bone regeneration, either on days 40 or 100 after surgery. Computed tomography, micro-computed tomography and chemical composition analyses were conducted on different bone tissues (cortical, trabecular and woven) at several skeletal locations (the operated metatarsus, the contralateral one and the iliac crest) to assess the individual bone quality changes relative to the non-osteoporotic time point.

**Results:**

After osteoporosis induction, the trabecular tissue experienced a 6.4% reduction in the bone mineral density, while no significant changes were reported in cortical tissue quality. During bone regeneration, the operated bone increased significantly the woven ossification whilst the cortical mineral density decreased by 18.7%. Simultaneously, an early deterioration in the microstructure and chemical composition of the trabecular bone was observed in the iliac crest, persisting over time in non-operated trabecular regions.

**Conclusions:**

Osteoporosis causes uneven degradation to trabecular tissue quality across different bone locations. Furthermore, the bone regeneration process via bone transport in osteoporotic subjects leads to a systemic skeletal disorder that further impairs the bone quality, surpassing the damage caused by osteoporosis alone. This impairment appears to be intensified by the pre-existing osteoporotic condition.

## Introduction

Osteoporosis is the most common bone disease, affecting approximately 500 million people worldwide [1]. This systemic skeletal disorder is characterized by microarchitectural deterioration of the trabecular bone tissue, decreased bone mineral density (*BMD*) and chemical compositional changes, leading to increased skeletal fragility and a higher risk of fractures. The clinical outcome of osteoporosis is often fractures from low-energy trauma, known as fragility fractures, which would not typically occur in healthy bones. Globally, 37 million fragility fractures occur annually, affecting one-third of women and one-fifth of men over the age of fifty [2–4]. These fractures result in significant economic cost and major health consequences for patients such as severe pain, dependency and disability. In this context, it has been shown that the risk of fragility fractures increases 5-fold during the first year following an initial fracture at the same or different skeletal sites [5], then gradually decreases from a factor of 2.7 after one year to 1.4 after ten years [6]. Numerous preclinical and clinical studies indicate that, during the bone regeneration, a significant systemic bone quality loss may occur throughout the skeleton, especially in osteoporotic patients, further aggravating the disease [7–11]. In addition, the bone regeneration process is frequently compromised by the poor osteogenic capability of osteoporosis [7,12], resulting in delayed healing, non-unions, bone deformities, chronic pain and even post-surgical complications such as infections [13–15]. In these challenging bone healing situations, distraction osteogenesis via bone transport is a recognized technique, clinically indicated due to its potential to promote bone healing [16–18]. This gold-standard orthopedic procedure consists of gradually displacing a bone fragment, along an osteotomized gap [16,19]. Despite its medical interest, there is a lack of knowledge about the skeletal bone quality alterations of applying such complex techniques in osteoporotic patients.

In clinical studies, dual energy X-ray absorptiometry (*DXA*) is the gold standard method for assessing osteoporosis and osteopenia due to its speed, clinical accessibility and low-radiation exposure. Nevertheless, this method is limited by its two-dimensional assessment of trabecular macroscale or apparent *BMD* and tends to overestimate *BMD* due to its poor differentiation between trabecular and cortical bone tissues [20,21]. In this context, there are alternative techniques for assessing bone quality that are less common for clinical applications due to their cost, their high levels of radiation or their invasive nature (e.g., bone biopsy extraction). However, these techniques are a great source of knowledge about bone quality in experimental research. In this regard, computed tomography (*CT*) and micro-computed tomography (*μCT*) are two widely used three-dimensional and high-resolution techniques for assessing, respectively, the macroscale *BMD* [22–25], the microscale *BMD*, and the microstructure of bone samples [26–28], thus offering a multiscale analysis of the bone mineral tissue. In addition, chemical composition analyses of bone samples can provide insights into the bone volumetric composition and stoichiometry of bone tissue as seen in studies on cortical and woven tissues of healthy subjects [29,30]. These techniques could enhance the understanding of bone regeneration in osteoporotic patients, aiding clinicians to develop novel patient-specific treatments and therapies. This could lead to a more effective medical care and follow-up of the disease, reducing recovery time and improving patients’ quality of life, as well as contributing to the development of preventive strategies focused on reducing the risk of refractures or secondary fractures.

Osteoporosis clinical and experimental research primarily focuses on understanding the biochemical and mechano-biological mechanisms underlying the disease, identifying risk factors [31–33], improving early detection techniques [1,34–39], developing better drug treatments or therapies for patients [40–43], or preventing the onset of the disease or fragility fracture through nutrition [44–46] or physical exercise [47,48]. A smaller proportion of this effort is dedicated to enhancing bone healing management. In this context, large animal models faithfully represent human bone conditions due to their size and anatomy, enhancing translational relevance and assessment of treatment efficacy in osteoporosis research. Numerous large animal studies have validated or characterized the effect of various osteoporosis induction protocols on bone quality [49–56], providing the temporal evolution data on *BMD* or microstructure of cortical or trabecular tissues, commonly by *DXA* and *μCT* [57–67]. For example, Zhang et al. [61] studied the influence of ovariectomy in sheep after 12 months from surgery by *μCT* of the trabecular tissue at the lumbar vertebra, femoral neck, mandibular angle and rib. They reported significant differences in trabecular microstructure degradation depending on the skeletal site location. Similarly, Bisazza et al. [57] compared *DXA* and *CT* techniques to assess temporal changes in trabecular and cortical *BMD* at lumbar vertebrae in the sheep osteoporotic model. They concluded that *CT* provides greater accuracy in detecting changes in *BMD* and bone microstructure. Nevertheless, few large animals’ studies have evaluated the skeletal bone quality evolution experienced by individuals adding the complexity of bone regeneration [10,11]. In this challenging context, Lill et al. [11] assessed by *CT* the macroscale *BMD* changes in cortical and trabecular tissues of the tibia between healthy and osteoporotic sheep groups after a mid-shaft tibia1 osteotomy. They found a significant apparent *BMD* downtrend, but without giving information about other bone locations or microstructure and chemical composition changes. In contrast, Bindl et al. [10] studied the influence of osteoporosis and right femoral metaphysis bone gap healing on different skeletal locations, providing insights into the microstructural bone quality loss in tibial cortical and trabecular tissues between healthy and osteoporotic sheep groups. However, there is a lack of knowledge regarding the temporal bone quality loss experienced by the subject-specific not only in terms of microstructure, but also in terms of mineral density and chemical composition, during the onset of osteoporosis and the subsequent bone regeneration process. Furthermore, as these studies present a simple fracture healing model, they do not provide information about how osteoporosis influences on bone regeneration processes of major complexity but frequently used in cases of severe trauma, such as the distraction osteogenesis model. In contrast, there are studies that have assessed different bone regeneration processes in healthy large animals, such as fracture healing [68–72] or distraction osteogenesis [73–77]. The woven bone generated within the callus has been characterized through different approaches like nanoindentation [78,79], biomechanical tests [11,80,81], histology [71,79,81,80], *CT* [11,73,75], finite element analysis from *CT* reconstructions [80,82] and gait analysis [74,75] among others. However, these studies lack information on the characterization of this immature tissue in osteoporotic subjects.

From all of this, it is essential to deepen understanding the skeletal bone quality changes in osteoporotic patients adding the complexity of bone regeneration. As a novelty, the present work will explore this combined condition through *in vivo* experiments using a large osteoporotic animal model undergoing a bone regeneration process treated by distraction osteogenesis via bone transport.

We hypothesize that the osteoporotic bone regeneration process significantly aggravates the systemic bone quality deterioration beyond the baseline effects of osteoporosis, which could be tested by measuring the mineral density, microstructure and chemical composition. In addition, the bone impairment may be intensified due to the presence of the disease. Thus, the study aims to elucidate and compare the bone quality changes in different bone locations of individuals during osteoporosis and osteoporotic bone regeneration. This will be achieved through multiscale imaging techniques (*CT* and *μCT*) and chemical composition analyses to explore temporal evolution of specific subject’s skeletons. In this sense, the macroscale and microscale *BMD*, microstructure, and chemical composition of different bone tissues (cortical, trabecular and woven bones) across different skeletal sites (the operated bone, its contralateral counterpart and a bone far from the operated bone) will be evaluated temporally and individually.

## Materials and methods

This section is organized as follows: firstly, the osteoporotic animal model and the distraction procedure using bone transport (*BT*) are described. Next, the study design is outlined to quantify the individual temporal impact of osteoporosis and the regeneration of osteoporotic bone defect across different bone locations and tissues. Finally, the methodology followed in the different analyses is described: the apparent or macroscale *BMD* (*BMD*_*CT*_) data measured by *CT*, the microscale *BMD* (*BMD*_*μCT*_) and microstructure data measured by *μCT*, and chemical composition analysis (ash fraction, volumetric composition, and elemental mass content).

### Osteoporotic animal model and distraction procedure

Osteoporosis was induced in a total of fifteen female merino sheep (weight 60.2 ± 5.6 kg), 2–4 years old using the protocol provided by Zarrinkalam et al. [54]. The animals were selected from a research farm and marked on the wool to avoid confounders. The randomly selection criteria ensures that the specimens are healthy and have proper vaccination and deworming protocols. The sample size was calculated to reduce the number of animals required to a relevant minimum, obtaining significant differences. For this purpose, the insights reported by Bindl et al. [10] was used as a reference, in which similar parameters were analyzed in the same osteoporotic animal model. The sheep were transported to the Clinical Veterinary Hospital of the University of Cordoba, where all *in vivo* experiments were conducted. This research center has spacious, fenced and partially roofed outdoor facilities, where the animals were housed and cared for. The induction period began with a bilateral ovariectomy. The ovariectomized sheep received periodic intramuscular injections of glucocorticoid (500 mg Solu-Moderín® + 7.8 ml injectable water) every 3 weeks for 33 weeks. Additionally, they were exclusively fed with a calcium-free diet till sacrifice, comprising 12% crude protein, 9% crude fiber, 6.5% crude ash, 2% crude fat, 0% calcium, 0.1% phosphorus and 0.1% sodium.

**Fig 1:**
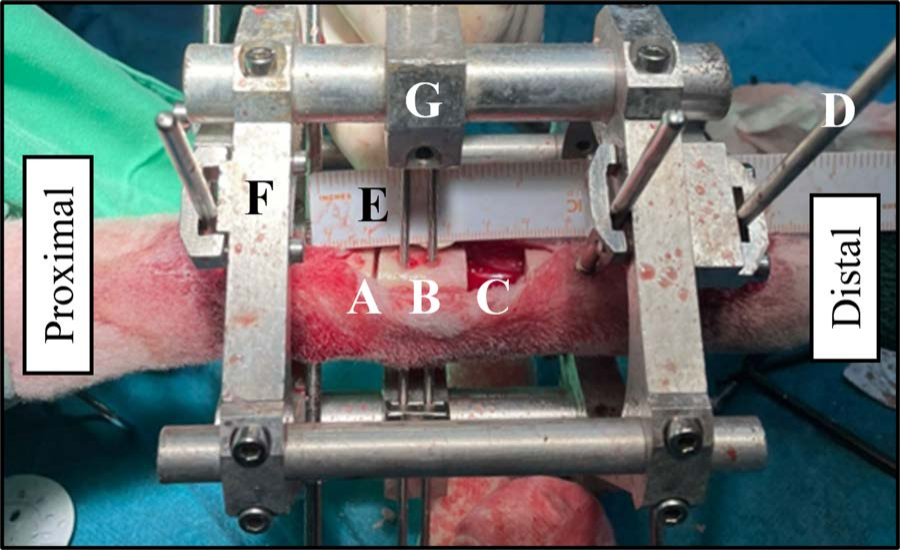
Bone transport (*BT*) surgery procedure in the right hind metatarsus of an ovariectomized sheep. Fixator implantation and bone defect performance: **(A)** proximal cross-sectional osteotomy; **(B)** 25 mm transport fragment; **(C)** 15 mm critical-size bone defect; **(D)** Ø4 mm Schanz-screws; **(E)** Ø2.5 mm Steinmann pins; **(F)** external fixator frames; **(G)** non-instrumented fixed bars.

The *BT* experiments began with a surgical procedure performed on the right hind metatarsus of the ovariectomized animal at week 33 ± 2.5. The sheep was under general anesthesia and intubated during both surgical procedures (*BT* surgery and ovariectomy), while the body temperature, blood pressure, oxygen levels, exhaled carbon dioxide levels, and electrocardiograms were continuously monitored. As illustrated in Fig 1, an Ilizarov-type external fixator [73–75,83] was initially implanted using Ø4 mm Schanz screws. Once the metatarsus was stabilized, three cross-sectional osteotomies were made using a guided oscillating saw (1.2 mm thickness). These osteotomies created two diaphyseal bone fragments: a 25 mm proximal transportable bone fragment (previously attached to the distractor through two Ø2.5 mm Steinmann pins) and a distal 15 mm bone segment which was removed to generate a critical-size bone defect (Fig 1). Following the bone surgery, the calcium free diet was continued throughout the experimental period, but steroid injections were ceased to preserve postsurgical animal welfare. After one-week latency period, a 15-days distraction phase was carried out, applying a 1 mm/day distal displacement of the transportable bone fragment per day along the 15 mm bone gap. In this way, the naïve tissue formed within the proximal osteotomy was elongated (distraction callus) while the tissue of the critical-size bone defect was compressed (docking site callus). Finally, the animals were randomly sacrificed by an overdose of sodium pentobarbital IV Euthasol® at two different time points during the osteoporotic bone regeneration, at day 40 (n = 5) or at day 100 (n = 5) after the *BT* surgery.

Animal welfare was guaranteed throughout the induction period, surgical interventions and experimental phases following the ARRIVE guidelines, European (63/2010/EU) and national (RD 53/2013) regulations on animal research. The animal ethics of this study was approved by University of Córdoba (Protocol Number: 2021PI/21).

### Study design at different bone locations

**Fig 2:**
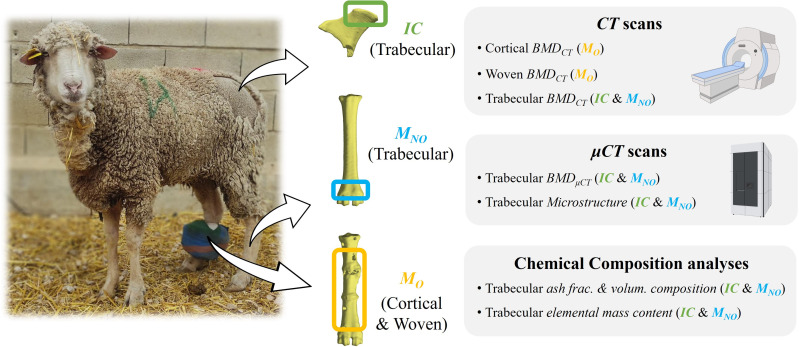
Study design scheme. Ovariectomized sheep analyses of the right hind metatarsus cortical and woven bone tissue ***(M***_***O***_**)**, the distal metaphysis trabecular tissue of the left hind metatarsus ***(M***_***NO***_**)**, and the iliac crest trabecular tissue (*IC*).

As shown in Fig 2, various bone tissue types from three bone locations of the sheep were analyzed: the right hind metatarsus (*M*_*O*_, cortical and woven tissues) as the operated bone, the distal left hind metatarsus metaphysis (*M*_*NO*_, trabecular tissue) as the contralateral counterpart, and the iliac crest (*IC*, trabecular tissue) as a bone distant from the operated bone. The bone samples were evaluated at different time points: on the day of ovariectomy as a non-osteoporotic time point (*Healthy*), on the day of the *BT* surgery (week 33 after the ovariectomy) representing the osteoporotic time point (*OP*), and on the day of sacrifice, 40 or 100 days after surgery, representing the osteoporotic bone regeneration time point (*OP + R40* or *OP + R100*, respectively). Table 1 shows the time points at which the samples were measured by the different analyses.

**Table 1:** Study design overview.

Bone assessment			Time points			
Name	Interest	Tissue type	*(a) Healthy*	*(b) OP*	*(c) OP + R40*	*(d) OP + R100*
Right Hind Metatarsus (*M*_*O*_)	Operated bone	Cortical & Woven	*CT*	*CT*	*CT*	*CT*
Left Hind Metatarsus (*M*_*NO*_)	Contralateral counterpart	Trabecular	*CT*	*CT*	*CT* *μCT* *ChC*	*CT* *μCT* *ChC*
Iliac Crest (*IC*)	Distant bone from the operated one	Trabecular	*μCT* *ChC*	*μCT* *ChC*	*CT* *μCT* *ChC*	*CT* *μCT* *ChC*

Different bone locations (*M*_*O*_, *M*_*NO*_ and *IC*) and bone tissue types (cortical, trabecular and woven) analyses (*CT*: computed tomography, *μCT*: micro-computed tomography and *ChC*: chemical composition) at each time point: *(a) Healthy*, on the day of ovariectomy; *(b) OP*, on the day of the *BT* surgery, 33 weeks after the ovariectomy; *(c) OP + R40* and *(d) OP + R100*, on day 40 and 100 after the *BT* surgery, respectively.

The influence of osteoporosis and osteoporotic bone regeneration on the bone type and location was evaluated with *BMD*_*CT*_ measurements in woven, cortical and trabecular bones by *CT*. And the analysis through scales and locations were assessed with *BMD*_*μCT*_, microstructure and chemical composition measurements of trabecular bone (see Table 1). For this purpose, *μCT* and chemical composition analyses were performed in trabecular biopsies extracted from *M*_*NO*_ and *IC*. *M*_*NO*_ biopsies were only extracted after sacrificing (*OP + R40* or *OP + R100*), as performing this procedure *in vivo* could compromise animal welfare. Meanwhile, the *CT* scans and the *IC* biopsy extractions were conducted *in vivo* for *Healthy* and *OP* time points during the surgical procedures (under general anesthesia and intubated), and *ex vivo* for *OP + R40* and *OP + R100* time points. Unlike the *M*_*NO*_, the *IC* as distant bone is suitable for *in vivo* biopsy extractions as it is easily accessible and without major harvesting risk, with abundant trabecular tissue. The biopsies were extracted using a biopsy punch and were preserved at -80ºC in PBS-soaked gauzes.

For the different analysis performed on all bone samples, each sheep data reported at the *Healthy* time point is used as its individual control data. In this way, each sheep’s data at subsequent time points were also normalized by its respective control data, thus providing an individualized temporal evolution of the animal.

### Macroscale data: *BMD*_*CT*_ using *CT*

**Fig 3:**
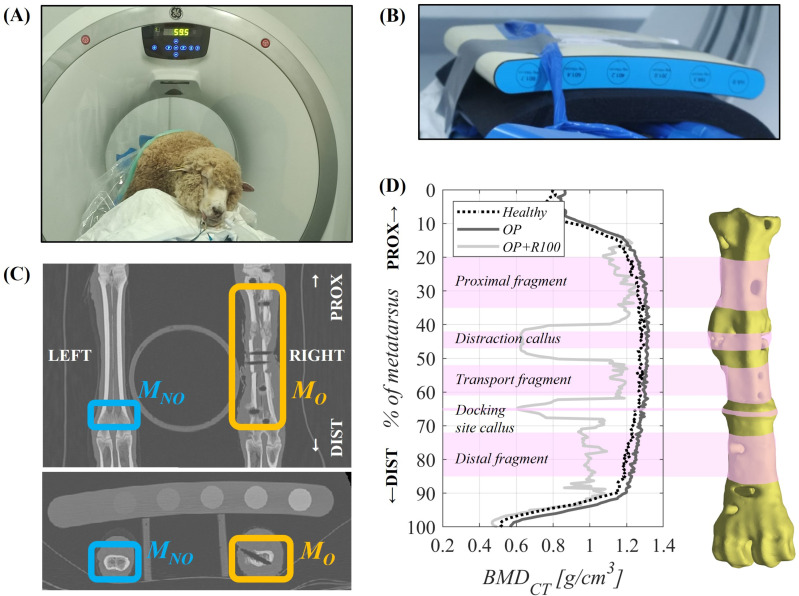
Macroscale *BMD* characterization of the osteoporotic bone. *CT* assessment of the *BMD*_*CT*_ in cortical (operated metatarsus, *M*_*O*_: proximal, transport and distal fragments), in woven (*M*_*O*_: distraction callus, docking site callus) and in trabecular (non-operated metatarsus, *M*_*NO*_). **(A)** *CT* measurement of an ovariectomized sheep. **(B)** *CT* phantom included in the *CT* measurement. **C)** *CT XZ* (top) and *XY* (down) measurement projections. **(D)** Cross-sectional *BMD*_*CT*_ throughout the bone distal percentage of *M*_*O*_ length (0% proximal, 100% distal) in an ovariectomized sheep at *Healthy*, *OP* and *OP + R100* time points.

As illustrated in Fig 3A, *CT* scans were performed *in vivo* on the animal under general anesthesia and intubated at the Clinical Veterinary Hospital of the University of Cordoba. All *CT* images were acquired using Revolution ACT (General Electric, Pekin, China) *CT* scanner (*XYZ* voxel size 460–570 x 460–570 x 625 μm/px). A *CT* phantom QRM-BDC/6–200® (PTW, Freiburg, Germany) was also included to linearly correlate *BMD*_*CT*_ (0–800 mg HA/cm^3^) with the stack’s Hounsfield Units (Fig 3B). All the bone samples (*M*_*O*_, *M*_*NO*_ and *IC*) and the phantom inserts (6 x Ø = 18 mm, h = 200 mm) were individually segmented using the open-source image processing tool ImageJ-Fiji. From each axial cross-sectional slice, a mean and standard deviation value of the *BMD*_*CT*_ was estimated at each time point. Intermediate *CT* scans were taken at weeks 10 and 20 after ovariectomy (*10w* and *20w* time points, respectively) to assess the temporal evolution of *BMD*_*CT*_ in *M*_*NO*_ during the osteoporosis induction phase. The trabecular tissue was segmented without including the cortical tissue in the *M*_*NO*_ (13% of total length of the left hind metatarsus) and *IC* (complete bone).

As shown in Fig 3D, different representative regions within the operated metatarsus (*M*_*O*_) were monitored to quantify the local and temporal evolution of the *BMD*_*CT*_ in the cortical and woven bone tissues according to each animal’s final *CT* scan (*OP + R40* or *OP + R100*). Mean and standard deviation values of *BMD*_*CT*_ were calculated from three cortical fragments and two bone calluses. The cortical *BMD*_*CT*_ was quantified in the proximal, transport and distal fragments (13%, 9% and 12% of the total length of *M*_*O*_, respectively). The woven bone *BMD*_*CT*_ was measured in the distraction and docking site calluses (5%, and 0.5% of the total length of *M*_*O*_, respectively).

### Microscale data: *BMD*_*μCT*_ and microstructure using *μCT*

**Fig 4:**
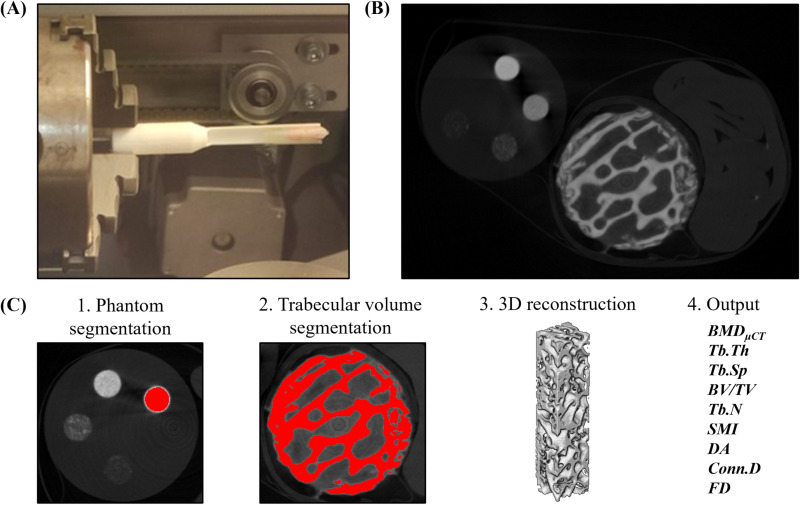
Microscale *BMD* and microstructure characterization of the osteoporotic trabecular tissue samples. **(A)** *μCT* set up. (B) *XY μCT* stack: *IC* biopsy and *μCT* phantom fixed to a 3D printed support (top). **(C)** *μCT* measurement procedure.

The *μCT* measurements were taken with the *μCT* scanner (*XYZ* voxel size 22.17–34.61 μm/px) model Y. COUGAR SMT (YXLON, Hamburg, Germany) at the University of Seville Research, Technology and Innovation Center (CITIUS). As shown in Figs 4A and 4B, the *μCT* measurement includes harvested trabecular biopsies from the iliac crest (*IC*) or contralateral metatarsus (*M*_*NO*_), along with a cylindrical *μCT* phantom, both fixed to a 3D printed support and placed within the *μCT* scanner. The *μCT* phantom (Ø = 4.5 mm, h = 5 mm) model MicroCT-HAD4.5® (QRM, Moehrendorf, Germany) was included to establish a linear correlation between the *BMD*_*μCT*_ of the phantom inserts (0–1200 mg HA/cm³) and the Hounsfield Units of the *μCT* stack. The *μCT* measurements were carried out using an open *μCT* multifocus tube of 25–160 kV voltage and current intensity of 0.01–1 mA.

The complete post-processing methodology for the *μCT* measurements is illustrated in Fig 4C, according to the guidelines for assessment of bone microstructure provided by Bouxsein et al. [84]. First, the *BMD*_*μCT*_ of the phantom inserts (5 x Ø = 0.8 mm, 4.5 mm height) was measured using ImageJ-Fiji® by means of the plugging BoneJ. Using the Amira-Avizo® software, all trabecular samples were segmented with a lower threshold of 400 mg HA/cm^3^ for normalized segmentation value of Hounsfield Units. The threshold was conﬁrmed by visual inspection after checking its capability to adequately discriminate between trabeculae and other components presented in the different bone samples (pores, blood, air, etc.). From the 3D reconstruction of the trabecular volume, the trabecular microstructural parameters were calculated: the trabecular thickness (*Tb.Th*), the trabecular separation (*Tb.Sp*), the bone volume per total volume (*BV/TV*), the trabecular number (*Tb.N*), and the structure model index (*SMI*). Finally, the degree of anisotropy (*DA*), the connectivity density (*Conn.D*), the fractal dimension (*FD*), and the mean cross-sectional microscale *BMD*_*μCT*_ value of the sample was measured using ImageJ-Fiji® by means of the plugging BoneJ.

### Chemical composition analyses

Chemical composition analyses were performed on the trabecular biopsies from the iliac crest (*IC*) and the contralateral metatarsus (*M*_*NO*_) after the *μCT* measurements at the University of Seville Research, Technology and Innovation Center (CITIUS). Firstly, a manual grinding procedure was carried out using a sterilized pestle and mortar to reduce the samples to a particle size of 1 mm. Subsequently, the total mass *m*_*t*_ of the sample was measured. This mass comprises the mass of water *m*_*w*_, the organic mass or organic phase *m*_*o*_ and the mineral mass or mineral phase *m*_*m*_, as defined in Eq. 1.


$$m_{t}=m_{w}+m_{o}+m_{m}$$ (1)

Afterwards, a drying procedure was applied to the sample by heating it at 105ºC for 1 hour in a BINDER VD 23 vacuum drying chamber (BINDER GmbH, Tuttlingen, Germany). The samples were weighed and heated again at 105ºC every 15 minutes until a constant dry mass *m*_*d*_ was achieved.


$$m_{d}=m_{o}+m_{m}=m_{t}-m_{w}$$ (2)

Then, the samples underwent an ashing process in a Nabertherm Mufﬂe Furnace (Nabertherm GmbH, Lilienthal Germany), following the protocol provided by Martínez-Reina et al. [29]: (A) a lineal increase to 250ºC for 30 min; (B) a constant temperature of 250ºC for 1 hour; (C) a lineal increase from 250ºC to 650ºC for 30 min; (D) a constant temperature of 650ºC for 2 hours; (E) sample weighing and 650ºC constant temperature for 30 min until a constant ash mass *m*_*a*_ is achieved. The protocol burns the organic phase of the sample, so *m*_*a*_ corresponds with the mineral phase, as shown in Eq. 3.


$$m_{a}=m_{m}=m_{d}-m_{o}$$ (3)

To assess the mineral content, the ash fraction, *α*, was calculated using Eq. 4.


$$\alpha =\frac{m_{a}}{m_{d}}=\frac{m_{m}}{m_{m}+m_{o}}$$ (4)

The volumetric fractions *v*_*x*_ of the mass *x* is obtained by:


$$\nu_{x}=\frac{V_{x}}{V_{w}+V_{o}+V_{m}}=\frac{\frac{m_{x}}{\rho_{x}}}{\frac{m_{w}}{\rho_{w}}+\frac{m_{o}}{\rho_{o}}+\frac{m_{m}}{\rho_{m}}}$$ (5)

where the density of the mass is *ρ*_*w*_ = 1 g/cm^3^, *ρ*_*o*_ = 1.43 g/cm^3^ [85] and *ρ*_*m*_ = 3.12 g/cm^3^ [29].

Later, an elemental analysis was carried out using a TruSpec Chns Micro analyzer (LECO Corporation, St Joseph, MI, USA) to determine the mass percentage of carbon. After previously dissolving the samples in hydroalcoholic acid, the mass percentage of calcium, phosphorus, magnesium, potassium, sodium and strontium were finally acquired through inductively coupled plasma atomic emission spectrometer Ultima 2 (HORIBA Jobin Yvon, Edison, NJ, USA).

### Statistical analyses

The statistical analyses were carried out using the software tool MATLAB R2023b® (The MathWorks Inc., Natick, MA, USA). A mean and standard deviation value from the individuals was obtained for all parameters measured at every time group (*Healthy*, *OP*, *OP + 40* and *OP + R100*) in each analysis (*CT*, *μCT* and chemical composition). Simultaneously, a mean and standard deviation value was calculated from the individual data previously normalized by its respective *Healthy* time point. The underlying absolute and normalized individual data are compiled in the S1-S4 Tables. The time groups outliers were checked and excluded using a Grubbs’ test [86] when *p* < 0.05. A Shapiro–Wilk test was performed to verify the normality of time groups. The data were analyzed in search of significant differences among time groups using non-parametric tests due to the non-normal distribution (*p* < 0.05) of the time groups presented in the different analyses (absolute and normalized data). Kruskal-Wallis test followed by Dunn–Sidak post hoc and correction was performed in every analysis of more than two-time groups. Meanwhile, Mann-Whitney U test was applied in the analyses when two-time groups were only analyzed. The tests have been selected considering the samples as unpaired as all of them are not exactly the same individuals (*OP + R40* versus *OP + R100*) or samples, or they have been altered in the different time groups. The p-values for the significance among time groups are compiled in the S5-S8 Tables.

## Results

### Impact of osteoporosis and osteoporotic bone regeneration on the bone tissue type

**Fig 5:**
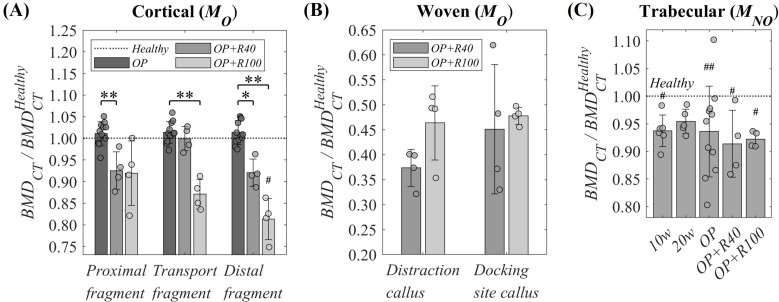
Individual temporal evolution of macroscale BMD in osteoporotic hind metatarsi. **(A)** Cortical *BMD*_*CT*_ in *M*_*O*_: proximal, transport and distal fragments. **(B)** Woven *BMD*_*CT*_ in *M*_*O*_: distraction callus and docking site callus. **(C)** Trabecular *BMD*_*CT*_ in *M*_*NO*_. Data normalized to the *Healthy* time point (compiled in S1 and S2 Table) and presented as mean ± standard deviation values. Significant differences among time groups: * means *p* < 0.05, ** means *p* < 0.01 and *** means *p* < 0.001. Significant differences of one time point versus *Healthy* time point: # means *p* < 0.05, ## means *p* < 0.01 and ### means *p* < 0.001. Significance evaluated by Kruskal-Wallis test followed by Dunn–Sidak post-hoc and correction for cortical and trabecular tissue and Mann-Whitney U test for woven tissue. Statistical analyses compiled in S5 and S6 Tables.

The temporal evolution of the *BMD*_*CT*_ in the cortical, woven, and trabecular bone tissues are presented in Tables 2 and 3. These data normalized to the individual *Healthy* time point value are also represented in Figs 5A-5C. The underlying data are presented in S1 and S2 Tables, while the respective statistical analysis is shown in S5 and S6 Tables.

**Table 2:** Macroscale *BMD* characterization in the osteoporotic operated bone.

*M*_*O*_ tissue	*M*_*O*_ region	Time points			
		*(a) Healthy*	*(b) OP*	*(c) OP + R40*	*(d) OP + R100*
**Cortical**	**Proximal fragment**	1.244 ± 0.054	1.268 ± 0.042^*c,d*^	1.126 ± 0.054^*b*^	1.175 ± 0.076^*b*^
	**Transport fragment**	1.265 ± 0.042^*d*^	1.289 ± 0.031^*d*^	1.233 ± 0.043	1.120 ± 0.040^*a,b*^
	**Distal fragment**	1.211 ± 0.046^*d*^	1.241 ± 0.045^*d*^	1.088 ± 0.062	0.983 ± 0.081^*a,b*^
**Woven**	**Distraction callus**	1.273 ± 0.043 (cortical)	–	0.444 ± 0.049	0.606 ± 0.089
	**Docking site callus**	1.246 ± 0.046 (cortical)	–	0.519 ± 0.145	0.599 ± 0.022

^a,b,c,d^p<0.05.

*BMD*_*CT*_ [g/cm^3^] measured at the operated metatarsus (*M*_*O*_) in three cortical fragments (proximal, transport and distal fragments) and in two woven bones (distraction callus and docking site callus) at four time points: *(a) Healthy*, on the day of ovariectomy; *(b) OP*, on the day of the *BT* surgery, 33 weeks after the ovariectomy; *(c) OP + R40* and *(d) OP + R100*, on day 40 and 100 after the *BT* surgery, respectively. Data expressed as *BMD*_*CT*_ mean ± standard deviation. Significance evaluated by Kruskal-Wallis test followed by Dunn–Sidak post-hoc and correction for cortical tissue and Mann-Whitney U test for woven tissue. ^*x*^ means significant differences (*p* < 0.05) in mean and standard deviation values of one time point versus other time point (*x*). Underlying data and statistical analyses compiled in S1 and S5 Tables, respectively.

**Table 3:** Macroscale *BMD* characterization in the osteoporotic contralateral bone.

Trabecular *M*_*NO*_ time points					
*(a) Healthy*	*(b) 10w*	*(c) 20w*	*(d) OP*	*(e) OP + R40*	*(f) OP + R100*
0.4643 ± 0.0552	0.4512 ± 0.0536	0.4317 ± 0.0523	0.4211 ± 0.0568	0.4128 ± 0.0321	0.4544 ± 0.0475

*BMD*_*CT*_ [g/cm^3^] measured in the trabecular non-operated hind metatarsus (*M*_*NO*_) at six time points: *(a) Healthy*, on the day of ovariectomy; *(b) 10w*, 10 weeks after the ovariectomy; *(c) 20w*, 20 weeks after the ovariectomy; *(d) OP*, on the day of the *BT* surgery, 33 weeks after the ovariectomy; *(e) OP + R40* and *(f) OP + R100*, on day 40 and 100 after the *BT* surgery, respectively. Data expressed as *BMD*_*CT*_ mean ± standard deviation. Significance evaluated by Kruskal-Wallis test followed by Dunn–Sidak post-hoc for cortical tissue and Mann-Whitney U test for woven tissue. ^*x*^ means significant differences (*p* < 0.05) in mean and standard deviation values of one time point versus other time point (*x*). Underlying data and statistical analyses compiled in S2 and S6 Tables, respectively.

Fig 5A shows the mean and standard deviation of the individual temporal evolution of the *BMD*_*CT*_ in the different cortical bone regions within the operated metatarsus (*M*_*O*_): proximal, transport and distal fragments. No significant differences were observed between the *Healthy* and *OP* time points. However, a significant decrease in *BMD*_*CT*_ was observed during the osteoporotic bone regeneration process when comparing the bone regeneration time points (*OP + R40* and *OP + R100*) with the *BT* surgery time point (*OP*). This reduction increases over time in the distal direction of the metatarsus, with *BMD*_*CT*_ loss of 20% of in distal fragment and 8% in proximal one at *OP + R100* relative to *Healthy*. This reduction of cortical *BMD*_*CT*_ is more significant on day 100 post-*BT* surgery than on day 40.

Regarding the woven bone tissue_,_ Fig 5B shows the individual temporal evolution of *BMD*_*CT*_ in the distraction and docking site bone calluses, relative to the mean cortical *BMD*_*CT*_ measured in the corresponding cortical region of the metatarsus at the *Healthy* time point. During the osteoporotic bone regeneration, a *BMD*_*CT*_ increase was observed in the distraction callus, with mean values of 37% at *OP + R40* and 46% at *OP + R100* relative to *Healthy* measures. The docking site callus *BMD*_*CT*_ was also increased, with mean values of 45% at *OP + R40* and 48% at *OP + R100*, and a reduction in the standard deviation over time.

Fig 5C illustrates the relative individual temporal *BMD*_*CT*_ evolution of the contralateral distal trabecular tissue. A progressively significant *BMD*_*CT*_ loss is observed throughout the osteoporotic induction period, reaching 6.4% relative to *Healthy* value at the *BT* surgery (*OP*). Then, this reduction is stabilized and remains constant during the bone regeneration process, reaching 8.6% and 7.8% *BMD*_*CT*_ loss at *OP + R40* and at *OP + R100*, respectively.

### Impact of osteoporosis and osteoporotic bone regeneration through scales and locations

**Fig 6:**
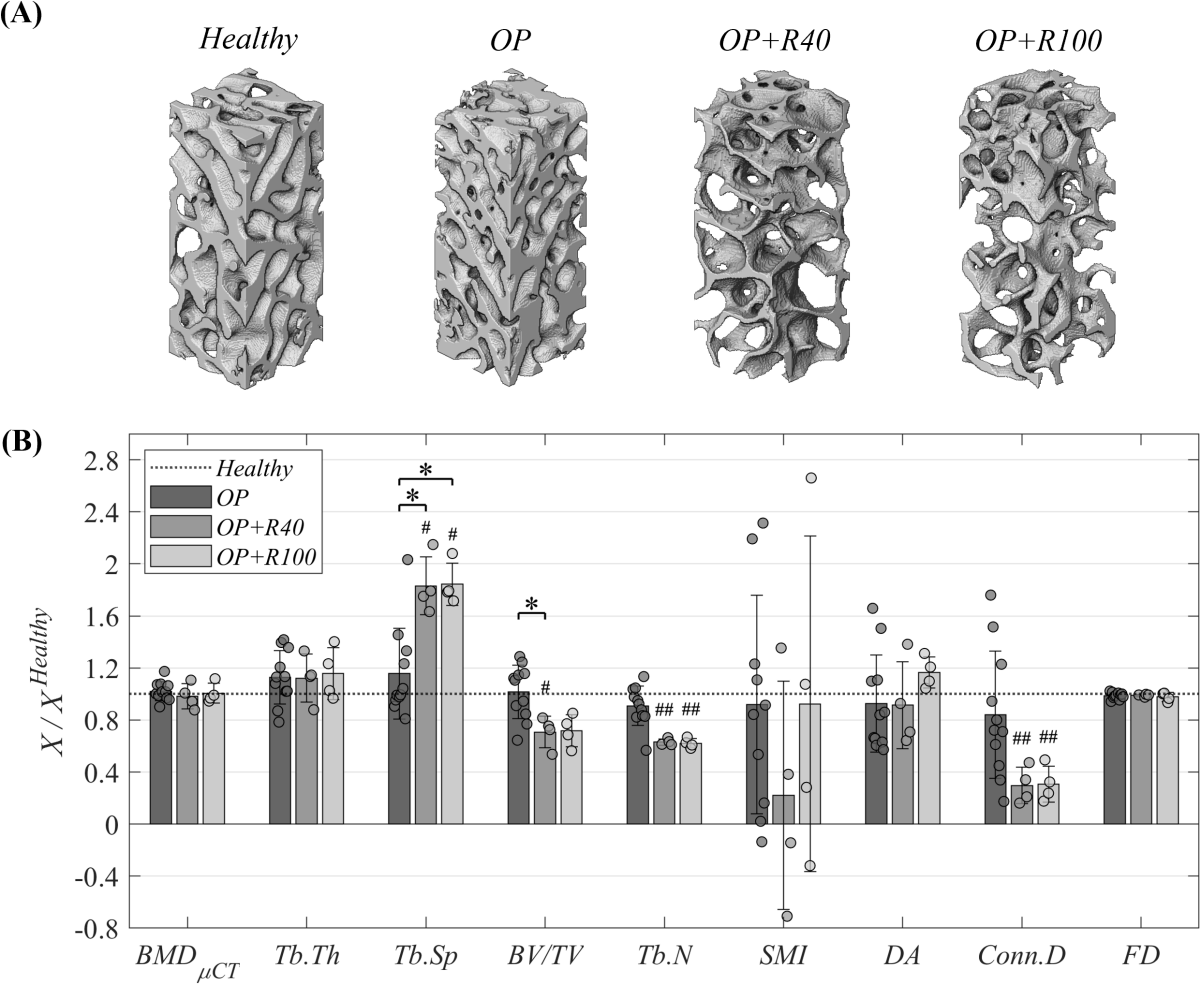
Microscale characterization of osteoporotic trabecular iliac crest (*IC*) biopsies. **(A)** 3D reconstruction comparison based on *μCT*, rendered using Amira-Avizo® software. **(B)** Individual temporal evolution of microscale *BMD* and microstructure. Data normalized to the *Healthy* time point (compiled in S3 Table) and presented as mean ± standard deviation values. Significant differences among time groups: * means *p* < 0.05, ** means *p* < 0.01 and *** means *p* < 0.001. Significant differences of one time point versus *Healthy* time point: # means *p* < 0.05, ## means *p* < 0.01 and ### means *p* < 0.001. Significance evaluated by Kruskal-Wallis test followed by Dunn–Sidak post-hoc and correction. Statistical analyses compiled in S7 Table.

To analyze the impact of osteoporosis and the osteoporotic bone regeneration through scales (macroscale and microscale) and locations, firstly the microscale data are described. The mean and standard deviation values of the temporal evolution of the *BMD*_*μCT*_ and the microstructure parameters in the trabecular iliac crest (*IC*) biopsies and contralateral metatarsus (*M*_*NO*_) are presented in Table 4. The *IC* data are normalized with respect to the *Healthy* time point value in Fig 6B. The underlying data are presented in S3 Table, while the respective statistical analysis is shown in S7 Table.

**Table 4:** Microscale *BMD* and microstructure characterization in osteoporotic non-fractured trabecular bones.

Measured Parameter	*IC*				*M*_*NO*_	
	*(a) Healthy*	*(b) OP*	*(c) OP + R40*	*(d) OP + R100*	*(e) OP + R40*	*(f) OP + R100*
***BMD***_***μCT***_ **[g/cm**^**3**^**]**	0.725 ± 0.034	0.742 ± 0.043	0.689 ± 0.042	0.709 ± 0.055	0.710 ± 0.038	0.746 ± 0.044
***Tb.Th* [µm]**	129.65 ± 21.45	141.36 ± 23.16	131.48 ± 22.50	137.29 ± 14.78	183.09 ± 30.97	197.57 ± 32.66
***Tb.Sp* [µm]**	262.05 ± 33.01^*c,d*^	291.60 ± 61.48^*c,d*^	471.49 ± 37.59^*a,b*^	449.90 ± 90.63^*a,b*^	299.6 ± 18.8	273.8 ± 46.0
***BV/TV* [%]**	33.20 ± 5.44^*c*^	33.04 ± 5.74^*c*^	21.70 ± 2.04^*a,b*^	23.88 ± 4.84	37.82 ± 5.38	41.98 ± 4.72
***Tb.N* [mm** ^**-1**^ **]**	2.568 ± 0.205^*c,d*^	2.356 ± 0.326^*c*^	1.671 ± 0.161^*a,b*^	1.734 ± 0.266^*a*^	2.074 ± 0.071	2.153 ± 0.283
***SMI* [-]**	-0.439 ± 0.285	-0.323 ± 0.482	-0.073 ± 0.214	-0.126 ± 0.468	-1.016 ± 0.223	-1.335 ± 0.428
***DA* [-]**	0.479 ± 0.112	0.424 ± 0.121	0.439 ± 0.122	0.430 ± 0.093	0.828 ± 0.026	0.839 ± 0.044
***Conn.D* [mm** ^**-3**^ **]**	9.187 ± 3.177^*d*^	7.359 ± 3.219^*d*^	3.264 ± 1.433^*a*^	3.124 ± 1.213^*b*^	2.032 ± 0.895	1.278 ± 0.348
***FD* [-]**	2.6420 ± 0.0464	2.6061 ± 0.0672	2.5948 ± 0.0457	2.5969 ± 0.0881	2.7040 ± 0.0506	2.7196 ± 0.0820

^a,b,c,d^p<0.05.

Assessment of *BMD*_*μCT*_ and microstructural parameters of trabecular thickness (*Tb.Th*), trabecular separation (*Tb.Sp*), bone volume per total volume (*BV/TV*), trabecular number (*Tb.N*), structure model index (*SMI*), degree of anisotropy (*DA*), connectivity density (*Conn.D*) and fractal dimension (*FD*) measured in four time points (*a*, *b*, *c* and *d*) for the *IC* (iliac crest) and two time points (*e* and *f*) for the *M*_*NO*_ (contralateral metatarsus): *(a) Healthy*, on the day of ovariectomy; *(b) OP*, on the day of the *BT* surgery, 33 weeks after the ovariectomy; *(c* and *e) OP + R40* and *(d* and *f) OP + R100*, on day 40 and 100 after the *BT* surgery, respectively. Data expressed as mean ± standard deviation. Significance evaluated by Kruskal-Wallis test followed by Dunn–Sidak post-hoc and correction for *IC* and Mann-Whitney U test for *M*_*NO*_. ^*x*^ means significant differences (*p* < 0.05) in mean and standard deviation values of one time point versus other time point (*x*). Underlying data and statistical analyses compiled in S3 and S7 Tables, respectively.

In the *IC*, no significant differences were observed in any parameter measured during the osteoporotic induction period (*Healthy* to *OP*). In contrast, significant differences were reported when comparing the trabecular separation (*Tb.Sp*), the bone volume per total volume (*BV/TV*), the trabecular number (*Tb.N*) and the connectivity density (*Conn.D*) between the bone regeneration time point (*OP + R40* or *OP + R100*) with the *OP* time point. As shown in Figs 6A and 6B, there was a significant increase in the *Tb.Sp* (68.1%), and a significant decrease in the *BV/TV* (30.3%), in the *Tb.N* (28.3%), and in the *Conn.D* (53.9%) from the *OP* to the bone regeneration time point (*OP + R40* or *OP + R100*). Regarding the remaining measured parameters, no significant differences between time points were obtained for the *BMD*_*μCT*_, the trabecular thickness, the structure model index, the degree of anisotropy and the fractal dimension. In addition, no significant differences were found between *OP + R40* and *OP + R100* time points in any of the parameters measured.

**Fig 7:**
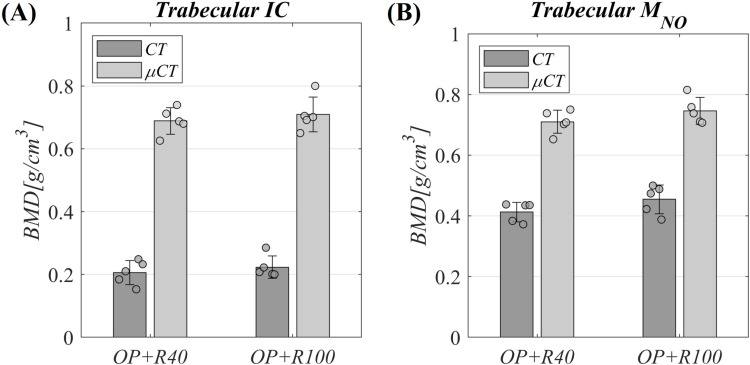
Multiscale *BMD* evaluation in osteoporotic non-fractured trabecular bones. Assessment at *OP + R40* and *OP + R100* time points, 40 and 100 days after the *BT* surgery, respectively. **(A)** Iliac crest (*IC*) assessment. **(B)** Non-operated metatarsus (*M*_*NO*_) assessment. Data presented as mean ± standard deviation values. * means *p* < 0.05, ** means *p* < 0.01 and *** means *p* < 0.001. Significance evaluated by Kruskal-Wallis test followed by Dunn–Sidak post-hoc and correction for *IC* and Mann-Whitney U test for *M*_*NO*_. Statistical analyses compiled in S6 and S7 Tables.

With respect to the previously mentioned impact through scales and locations, microscale *BMD* (*BMD*_*μCT*_) and macroscale *BMD* (*BMD*_*CT*_) are shown for the trabecular tissue from the *IC* and *M*_*NO*_ in Figs 7A and 7B, respectively (statistical analysis in S6 and S7 Tables, respectively). No significant *BMD* differences were found between both bone regeneration time points (*OP + R40* and *OP + R100*) for the trabecular bone locations assessed at both scales.

### Chemical composition characterization

**Fig 8:**
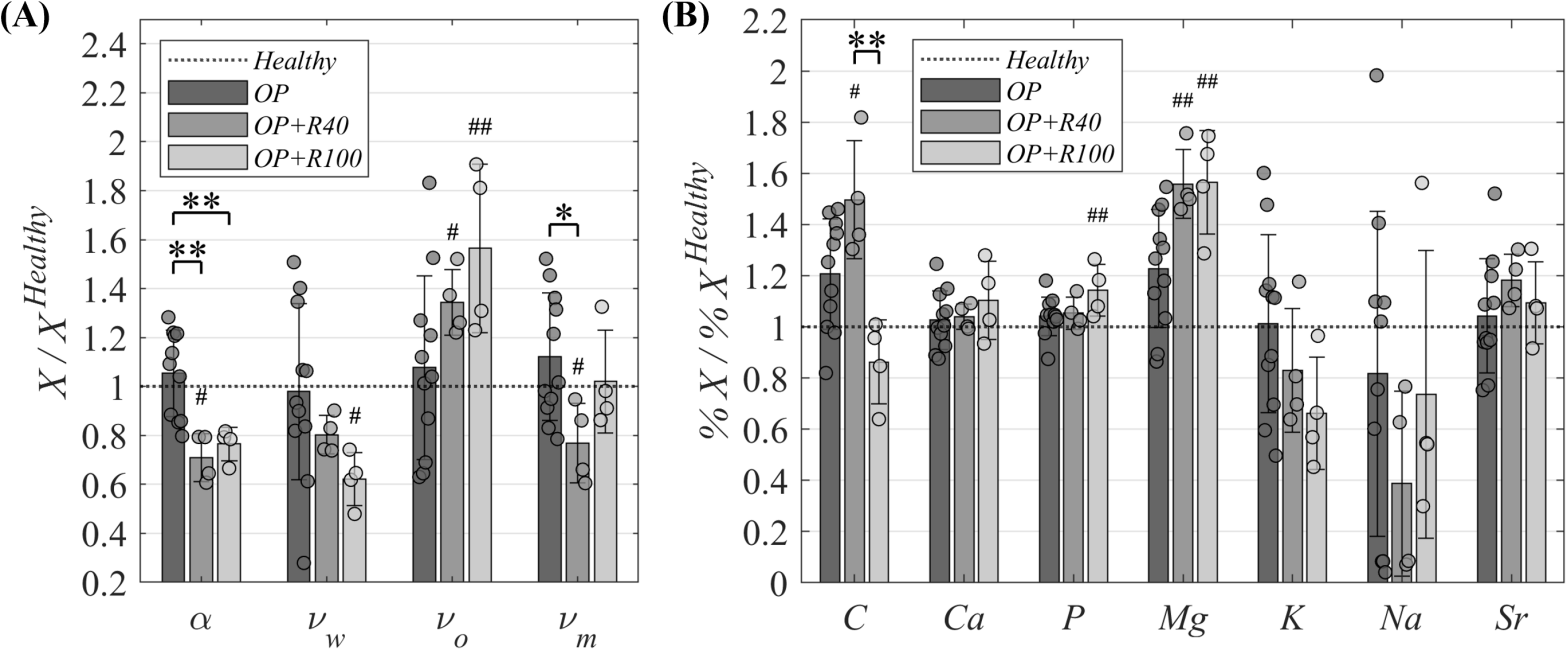
Individual temporal evolution of chemical composition in osteoporotic trabecular *IC* biopsies. **(A)** Individual temporal evolution of ash fraction (*α*), and volumetric composition of water (*ν*_*w*_), organic phase (*ν*_*o*_), and mineral phase (*ν*_*m*_). **(B)** Individual temporal evolution of the percentage of elemental mass content of C, Ca, P, Mg, K, Na, and Sr. Data normalized to the *Healthy* time point (compiled in S4 Table) and presented as mean ± standard deviation values. Significant differences among time groups: * means *p* < 0.05, ** means *p* < 0.01 and *** means *p* < 0.001. Significant differences of one time point versus *Healthy* time point: # means *p* < 0.05, ## means *p* < 0.01 and ### means *p* < 0.001. Significance evaluated by Kruskal-Wallis test followed by Dunn–Sidak post-hoc. Statistical analyses compiled in S8 Table.

The temporal evolution of the chemical composition analyses (ash fraction and volumetric composition, and the percentage of the elemental mass content) in the trabecular iliac crest (*IC*) biopsies and contralateral metatarsus (*M*_*NO*_) are shown in Table 5 as mean and standard deviation values. Both *IC* analyses are also presented relative to the individual *Healthy* time point values in Figs 8A and 8B. The underlying data are presented in S4 Table, while the respective statistical analysis is shown in S8 Table.

**Table 5:** Chemical composition characterization in osteoporotic non-fractured trabecular bones.

Analysis	Measured Parameter	*IC*				*M*_*NO*_	
		*(a) Healthy*	*(b) OP*	*(c) OP + R40*	*(d) OP + R100*	*(e) OP + R40*	*(f) OP + R100*
**Ash fraction & Volumetric composition**	***α* [-]**	0.4533 ± 0.0577	0.4653 ± 0.0536^*c,d*^	0.3412 ± 0.0624^*b*^	0.3415 ± 0.0741^*b*^	0.4256 ± 0.0326	0.4581 ± 0.0090
	***ν***_***w***_ **[-]**	0.4957 ± 0.0951	0.4598 ± 0.1305	0.4119 ± 0.1090	0.3182 ± 0.0797	0.2180 ± 0.0413	0.2503 ± 0.0254
	***ν***_***o***_ **[-]**	0.3668 ± 0.0835^*d*^	0.3884 ± 0.1103	0.4782 ± 0.1076	0.5527 ± 0.0976^*a*^	0.5840 ± 0.0471	0.5403 ± 0.0191
	***ν***_***m***_ **[-]**	0.1375 ± 0.0250	0.1518 ± 0.0282^*c*^	0.1099 ± 0.0066^*b*^	0.1291 ± 0.0197	0.1980 ± 0.0142	0.2094 ± 0.0090
**Elemental Mass Content**	**%C**	0.1860 ± 0.0459	0.2248 ± 0.0411	0.2237 ± 0.0615	0.2003 ± 0.0719	0.2360 ± 0.0445	0.2429 ± 0.0234
	**%Ca**	38.65 ± 2.57^*d*^	39.64 ± 2.12	39.78 ± 2.12	42.16 ± 0.96^*a*^	39.40 ± 2.58	37.08 ± 0.76
	**%P**	20.40 ± 1.16^*d*^	21.22 ± 1.25	21.60 ± 0.86	22.14 ± 0.77^*a*^	22.08 ± 1.01	21.19 ± 0.26
	**%Mg**	0.4613 ± 0.0493^*c,d*^	0.5663 ± 0.1117	0.7304 ± 0.1015^*a*^	0.6740 ± 0.0786^*a*^	0.7100 ± 0.0760	0.6591 ± 0.0621
	**%K**	0.4166 ± 0.1311	0.3928 ± 0.1123	0.3207 ± 0.0892	0.2976 ± 0.0715	0.1144 ± 0.0152	0.1326 ± 0.0303
	**%Na**	3.7869 ± 1.2736	3.3454 ± 2.0680	2.2662 ± 2.0841	2.1769 ± 0.5379	0.5734 ± 0.6193	0.6333 ± 0.6865
	**%Sr**	0.03452 ± 0.00728	0.03625 ± 0.01080	0.04005 ± 0.01705	0.03713 ± 0.00466	0.04208 ± 0.01179	0.04035 ± 0.00536

^a,b,c,d^p<0.05.

Ash fraction (*α*), and volumetric composition of water (*ν*_*w*_), organic phase (*ν*_*o*_) and mineral phase (*ν*_*m*_), and percentage of the elemental mass content analyses of the mineral phase of C, Ca, P, Mg, K, Na, and Sr measured in four time points (*a*, *b*, *c* and *d*) for the *IC* (iliac crest) and two time points (*e* and *f*) for the *M*_*NO*_ (contralateral metatarsus): *(a) Healthy*, on the day of ovariectomy; *(b) OP*, on the day of the *BT* surgery, 33 weeks after the ovariectomy; *(c* and *e) OP + R40* and *(d* and *f) OP + R100*, on day 40 and 100 after the *BT* surgery, respectively. Data expressed as mean ± standard deviation. Significance evaluated by Kruskal-Wallis test followed by Dunn–Sidak post-hoc and correction for *IC* and Mann-Whitney U test for *M*_*NO*_. ^*x*^ means significant differences (*p* < 0.05) in mean and standard deviation values of one time point versus other time point (*x*). Underlying data and statistical analyses compiled in S4 and S8 Tables, respectively.

For the *IC* evolution presented in Fig 8A, no significant differences were observed in the individual ash fraction and volumetric composition values during the osteoporotic induction period (between *Healthy* and *OP* time points). However, these parameters experienced different changes during the osteoporotic bone regeneration process. A significant reduction of up to 31.6% relative to *Healthy* value was reported for the ash fraction (*α*) with no significant differences between both bone regeneration time points (*OP + R40* and *OP + R100*). The water and organic phase volumetric fractions followed opposite trends, reaching a significant reduction of up to 35.7% in the volumetric fraction of water (*ν*_*w*_) and a significant increase of up to 48.8% in the volumetric fraction of organic phase (*ν*_*o*_) at the *OP + R100* time point. Regarding the volumetric fraction of the mineral phase (*ν*_*m*_), it showed a significant reduction of 45.4% relative to *Healthy* between *OP* and *OP + R40* time points, which recovered *Healthy* values at *OP + R100*.

Fig 8B shows the individual temporal evolution of the elemental mass content percentage of C, Ca, P, Mg, K, Na and Sr from the previously mentioned mineral phase of the *IC*. Table 5 shows the mass percentage of these elements, with Ca representing around 40%, P around 20%, and the sum of the other elements analyzed representing less than 6%. During the osteoporotic induction period, the mass percentage of C and Mg showed an increase of 20.7% and 22.8% relative to *Healthy* measurements, respectively. No changes were found in the remaining elements analyzed. During the osteoporotic bone regeneration process, the elements exhibited different trends, although the mass percentage of Na and Sr remained unchanged. The mass percentage of C significantly increased again by 29.0% at *OP + R40* (49.7% relative to *Healthy* value), but it returned to *Healthy* values at *OP + R100*. The mass percentage of Ca and P reported an increase of 10.14% and 14.23% at *OP + R100,* respectively, without differences in the early period of the osteoporotic bone regeneration (*OP + R40*). With respect to the mass percentage of Mg, it showed also a new significant increase of 33.34% during the osteoporotic bone regeneration (56.12% relative to *Healthy* value), without significant differences between both bone regeneration time points. Finally, the mass percentage of K reported a reduction trend of up to 35.02% at *OP + R100*, although without significant differences.

In addition, the osteoporotic trabecular bone comparison presented in Table 5 shows no significant differences in the ash fraction, volumetric composition, or elemental mass content between both osteoporotic bone regeneration time points (*OP + R40* and *OP + R100*) at both trabecular bone locations.

## Discussion

This *in vivo* study quantitatively assesses the individual temporal bone quality alterations due to osteoporosis and subsequent bone defect regeneration via bone transport in a large animal model. Different bone tissues (cortical, trabecular and woven) at different bone locations were evaluated by *CT*, *μCT* and chemical composition analyses. Specifically, three different osteoporotic bones were analyzed: the operated metatarsus with a critical-size bone defect under the regeneration process, its contralateral counterpart, and a bone far from operated bone, the iliac crest. Thus, this work covers, for the first time in the literature, the mineral, microstructural and chemical composition alterations suffered by the skeletal system of individual subjects with osteoporosis undergoing a complex bone regeneration process treated by distraction osteogenesis via bone transport.

Beginning with the macroscale analysis of the operated bone by *CT*, the results showed that the cortical *BMD*_*CT*_ of the operated metatarsus is not affected by 33 weeks of osteoporosis induction (*Healthy* to *OP* in Table 2 and Fig 5A). In contrast, the trabecular *BMD*_*CT*_ suffered a significant reduction at the distal contralateral counterpart (almost 7% relative to *Healthy* value) after the osteoporosis induction process (*Healthy* to *OP* in Table 3 and Fig 5C). These results are in line with previous findings in several studies in the same osteoporotic animal model [52,55,57]. Osteoporotic trabecular *BMD*_*CT*_ loss is associated with increased porosity, a hallmark of osteoporosis. Note that the apparent trabecular *CT* segmentation does not differentiate between trabeculae and pore phase. After the bone surgical intervention (*OP*), the distal contralateral trabecular tissue appears to be affected by the osteoporotic bone regeneration, reporting a non-significant reduction of *BMD*_*CT*_ (Fig 5C). During this period (*OP* to *OP + R40* or *OP + R100*), the woven tissue of the operated bone experienced an increased level of ossification (Table 2 and Fig 5B) while all cortical tissue samples (located at the proximal, transport and distal fragments) reported a reduction of the *BMD*_*CT*_. This decrease over time is more significant in the distal direction of the bone (Table 2 and Fig 5A). Particularly, the apparent cortical fragments *BMD*_*CT*_ decreased by 9.2% in proximal, 14.3% in transport, and 20% in distal fragments with respect to *Healthy* values after 100 days from *BT* surgery (*OP + R100*). Ren et al. [87] observed this early-stage cortical remodeling after osteotomy (63 and 84 days of bone regeneration) in healthy sheep tibia fracture healing model, with *BMD*_*CT*_ reductions of 1.3% and 2.2% at the proximal and distal bone fragments ends, respectively. The greater reductions in the current study are likely due to the higher size of our defect and the influence of induced osteoporosis. Augat et al. [68] proposed that this phenomenon might result from reduced load-bearing capacity in the operated limb following surgery, leading to bone tissue readaptation and resorption [74,83]. This condition induces a bone tissue readaptation in the intervened limb resulting in bone resorption. Another possible explanation that also could lead to an operated bone decreased osteoblastic activity, is the reduction of vascularization in the distal direction due to the osteotomies and the size of the defect [88]. This hypothesis can be supported by the more pronounced degradation of the cortical tissue in the distal direction of the metatarsus (Fig 5A). Augat et al. [68] also observed *BMD*_*CT*_ differences although not significant, probably due to the simplicity of the defect generated (2 mm osteotomy). One last hypothesis could be the need for bone mineral to repair the bone defects, which is in line to the mentioned increased level of ossification reported in both calluses at both bone regeneration, especially early in the docking site callus regeneration, as mechanically characterized by Mora-Macías et al. [78] in *BT* healthy sheep model. In the present study, the mentioned mineral need becomes even more critical due to the poor osteogenic capability caused by the pathology [7,12]. In this context, bone defect regeneration processes could produce a systemic skeletal disorder, making the areas closest to the defect a potential source of mineral resources to contribute to the bone regeneration [68]. However, the transport fragment does not seem to have a significant mineral contribution during the first 40 days after the bone intervention (*OP* to *OP + R40* in Table 2 and Fig 5A). This outcome is probably due to the vascular disconnection of this transport fragment which prevents its minerals from being used as a resource for both adjacent woven mineralization. In contrast, there was a significant loss of *BMD*_*CT*_ at 100 days after surgery (*OP* to *OP + R100*), suggesting the need for prior angiogenesis and woven bridging to contribute to both callus osteogenesis. Concluding with the macroscale characterization, in the operated bone, the significant cortical *BMD*_*CT*_ loss over time and woven mineralization indicates that the mineral has been mobilized for the bone defects regeneration treated by distraction osteogenesis. This need appears to be even more critical due to osteoporosis. In contrast, the influence of this disease alone in the *BMD*_*CT*_ seems to be limited to the trabecular tissue of the contralateral bone (Fig 5C).

Regarding the microscale analysis of the trabecular tissue performed in the iliac crest by *μCT* (Table 4 and Figs 6A and 6B), no significant differences were reported after 33 weeks of osteoporosis induction in any of the computed parameters (*Healthy* to *OP*). This finding supports previous studies indicating that osteoporosis does not uniformly affect trabecular tissues at different locations [89–91]. In contrast, the iliac crest trabecular microstructure was shown to remodel during the first 40 days after the *BT* surgery (*OP* to *OP + R40*), with a significant increase in the *Tb.Sp* (67%), and a decrease in the *BV/TV* (31%), in the *Tb.N* (28%), and in the *Conn.D* (54%) in relation to *Healthy* value. Consequently, the iliac crest trabeculae after the intervention are notably less dense and interconnected, with fewer trabeculae. As far as the authors are concerned, there are no studies assessing the individual microstructural changes of the trabecular tissue throughout a bone defect regeneration process in an osteoporotic sheep model to compare. However, our findings are in line with Bindl et al. [10], who compared osteoporotic and healthy sheep groups, the osteoporotic right femoral fracture healing also induces a decrease in the *BV/TV* of 32% and the *Tb.N* of 31%, and a 57% increase in the *Tb.Sp* at the right tibial trabecular tissue at 56 days post-fracture. In addition, the *μCT* analysis conducted by Fischer et al. [4] in osteoporotic mice revealed a significant degradation of trabecular microstructure (25% reduction in *BV/TV* and *Tb.N*) in the lumbar vertebrae of the femur fractured group compared to the non-fractured group. Regarding the *BMD*_*μCT*_ measured 40 days after *BT* surgery (*OP* to *OP + R40*), no significant differences were reported. Unlike *CT*, which computes an apparent *BMD* (*BMD*_*CT*_) with trabeculae and pores, *μCT* has the resolution to analyze the trabeculae focusing on the mineral phase, which appears to maintain the mean *BMD*_*μCT*_ while impairing the trabecular iliac crest microstructure. By 100 days post-surgery, the absence of significant differences in *BMD*_*μCT*_ and microstructure parameters of the iliac crest with respect to 40 days after surgery (*OP + R40* to *OP + R100*, 60 days difference) suggests that trabecular tissue far enough from the regeneration focus is not affected by the bone regeneration in an osteoporotic subject (Table 4 and Figs 6A and 6B). This is in line with the multiscale *BMD* results of both trabecular bones (Figs 7A and 7B) and the trabecular microstructure results of the contralateral metatarsus (Table 4). This microstructure was observed to be weaker (higher *Tb.Sp*, lower *Tb.N* and *Conn.D*) than that reported by Blázquez-Carmona et al. [77] in the operated metatarsus trabecular tissue of healthy sheep undergoing a bone lengthening process using distraction osteogenesis. According to this, the osteoclastic activity seems to increase in the osteoporotic trabecular tissue of far non-fractured bones at the early stage, during the first 40 days after the bone surgical intervention (*OP* to *OP + R40*). This fact is probably a systemic response of the osteoporotic skeleton to further contribute to the supply of minerals for the bone repair of the operated metatarsus. In addition, the main microscale changes in the trabecular tissue in the non-fractured bones are due to the combination of osteoporosis with the bone callus regeneration process, rather than solely osteoporosis.

In terms of chemical composition analyses of the iliac crest trabecular tissue, the ash and volumetric composition temporal evolution results are illustrated in Table 5 and Fig 8A. As far as the authors know, there are no studies in which the volumetric composition is assessed during osteoporosis and osteoporotic bone regeneration (the same for the ash fraction) at the non-fractured trabecular tissue to compare. Considering the differences, the results could be compared with those of Martínez-Reina et al. [29], who analyzed the volumetric composition and elemental mass content percentage of the lamellar (cortical) and woven tissue from the metatarsus of healthy sheep undergoing a *BT* regeneration process. According to Table 5 and Fig 8A, the parameters measured did not report significant changes at the end of the osteoporosis induction (*Healthy* to *OP*), consistent with Bloebaum et al. [92], who found no differences in the ash fraction between young and osteoporotic women groups at different trabecular sites. During the first 40 days after the bone surgical intervention (*OP* to *OP + R40*), a significant loss of the ash fraction took place (31.6% with respect to *Healthy* value). With respect to the volumetric composition of the iliac crest during the bone regeneration (*OP* to *OP + R40* or *OP + R100*), the significant opposite trends reported in water volumetric fraction (downtrend) and organic phase volumetric fraction (uptrend) seems to indicate a volumetric change comparable to that reported by Martínez-Reina et al. [29] in healthy woven bone. In this immature bone tissue, a high volumetric fraction of organic phase means that it is undergoing a process of mineral change, either absorption or resorption. In addition, a mineral phase loss of volumetric fraction occurred in the early period of osteoporotic bone regeneration, which appears to recover over time. All this information seems to indicate that the osteoporotic bone regeneration stimulates the production of organic matrix in distant non-fractured bones, which suggest the displacement of blood volume to contribute with the inflammation and the transport of minerals. This early loss of mineral phase mass in relation to the mineral phase plus organic phase mass (ash fraction) and water volumetric fraction reflects, once again, a mineral mass resorption and mobilization from the trabecular tissue of the iliac crest to contribute to the osteoporotic regeneration of the operated metatarsus. This hypothesis is consistent with the trabecular iliac crest microstructure parameters degradation discussed above (Table 4 and Figs 6A and 6B).

The percentage of the elemental mass content from the mineral phase of the iliac crest trabecular tissue is shown in Table 5 and Fig 8B. While no direct comparisons are available in the literature, Martínez-Reina et al. [29] measured the mass percentages of C, P, and Ca in non-osteoporotic cortical and woven tissue. The comparisons between this study and the current work are limited due to the osteoporosis influence and the differences between these bone tissues with the trabecular tissue, without also analyzing other elements such as Mg, K, Na and Sr. As shown in Fig 8B, the percentage of elemental mass content of C and Mg experienced an increase during the osteoporosis induction (*Healthy* to *OP*). An increase in the mass percentage of C (probably from a degradation of the organic matrix) and Mg, indicates an increase in the carbonate content of the hydroxyapatite, which may make it less stable and more fragile and susceptible to an osteoporotic increased reabsorption. However, the elemental mass percentages of Ca, P, K, Na and Sr remained constant, showing that in this trabecular bone the percentage of hydroxyapatite has not changed significantly due to osteoporosis. The most abundant metallic elements in the mass percentage of hydroxyapatite (Ca and P) appear not to change significantly during the osteoporotic bone regeneration (*OP* to *OP + R40* or *OP + R100*) of the operated metatarsus. However, it cannot be assumed that the mineral mass is not being lost, since the results are described as mass percentage. The same applies to three of the less abundant elements in the bone mineral, C, Na and Sr. In contrast, the mass percentage of K and Mg reported a significant decreasing and increasing trend, respectively, during the osteoporotic bone regeneration process, not reported during the osteoporotic induction period. This fact suggests a metabolic and homeostatic adaptation through the redistribution of secondary elements. In this regard, the increase in Mg could improve the formation of new bone tissue and cellular activity [93], while the decrease in K could indicate its less direct involvement in mineralization, thus focusing on the needs of osteoporotic bone regeneration of the operated bone. With respect to the comparison of the osteoporotic bone regeneration points for both trabecular tissues of non-fractured bones (iliac crest and contralateral metatarsus) in all the analyses (*CT*, *μCT* and chemical composition) performed in this work (Tables 4 and 5), no significant differences were found. This fact may indicate that both trabecular tissues have behaved in a similar way between days 40 and 100 after the bone surgery (*OP + R40* to *OP + R100*), prioritizing the osteogenesis of operated bone woven tissues over the recovery of non-fractured trabecular osteoporotic tissue.

As for the limitations of the work, it should be noted that not all the analyses presented have been carried out in all the bone tissues and locations considered throughout all the time points (*Healthy*, *OP*, *OP + R40* or *OP + R100*) of measurement of the sheep. In the case of *μCT* scanning and chemical composition analysis, the individual temporal evolution is only completed at the iliac crest, as any *in vivo* biopsy extractions at the operated or contralateral metatarsus would have compromised the welfare of the animals. In addition, two sheep suffered health difficulties and had to be slaughtered before the *BT* surgery. Meanwhile, three animals experienced post *BT* surgery complications that made it necessary to slaughter them before the bone regeneration time point. Thus, these specimens did not provide the complete individual temporal evolution data. The *CT* scans were limited to a single time point during bone regeneration due to the presence of metal elements in the implanted external fixator, which required post-sacrifice *CT* scans after fixator removal. Consequently, the comparison between the *OP + R40* and *OP + R100* time points refer to animals from different sacrifice groups, therefore it is not possible to talk about an individual temporal evolution when comparing both time points. Finally, the influence of osteoporosis during the bone regeneration process could not be determined explicitly since the study does not consider a healthy control group undergoing the same bone regeneration process.

In conclusion, this study provides novel quantitative insights into the alterations in bone quality experienced by individual subjects during the onset of osteoporosis combined with bone regeneration processes treated by distraction osteogenesis. Specifically, multiscale *BMD*, microstructure and chemical composition were assessed in different bone tissues and locations in osteoporotic large animal subjects undergoing a critical-size bone regeneration process. It is determined firstly that osteoporosis alone only seems to significantly impair the bone quality of trabecular tissue heterogeneously depending on the bone localization. Secondly, the osteoporotic bone regeneration process following bone transport interventions results in a systemic skeletal disorder, which is greater than osteoporosis itself, and appears to be intensified by this disease. At the operated bone, this combined condition caused a significant loss of cortical bone quality that was aggravated over time in the distal direction. In addition, the trabecular tissues of far bones were also especially affected during the early stage of bone regeneration, deteriorating their microstructure and altering their chemical composition. This generalized skeletal bone quality impairment seems to be a quick response of the organism to regulate the blood mobilization of bone mineral sources that can contribute to enhance the woven tissue mineralization of the operated bone. This mineral requirement is even more critical in osteoporotic individuals in whom the osteogenic capacity is severely diminished by estrogen deficiency, accelerating bone resorption and decreasing osteoblastic activity.

In this sense, the present work reflects the need to study the bone quality by different measurement approaches (multiscale *BMD*, microstructure, and chemical composition) at different bone locations to evidence the aggravation of the osteoporotic bone regeneration process. In osteoporotic patients who need distraction osteogenesis procedure, it is important to prioritize the continuous monitoring of the bones statistically more prone to a new fracture, especially the already threated bone under regeneration, whose fixation is at high risk of being mechanically and fatally compromised. Therefore, it is crucial the continuous bone quality care and clinical follow-up, especially during the early stage of bone regeneration, to ensure adequate healing, considering the several risks of refractures or secondary fractures.

## Supporting information

S1 Table Underlying data points of Table 2. Sheep data points of the macroscale BMD characterization in osteoporotic operated bone.
(XLSX)

S2 Table Underlying data points of Table 3. Sheep data points of the macroscale BMD characterization in osteoporotic non-fractured trabecular bones.
(XLSX)

S3 Table Underlying data points of Table 4. Sheep data points of the microscale BMD and microstructure characterization in osteoporotic non-fractured trabecular bones.
(XLSX)

S4 Table Underlying data points of Table 5. Sheep data points of the chemical composition characterization in osteoporotic non-fractured trabecular bones.
(XLSX)

S5 Table Statistical analyses of Table 2. P-values of the macroscale BMD characterization in osteoporotic operated bone.
(XLSX)

S6 Table Statistical analyses of Table 3. P-values of the macroscale BMD characterization in osteoporotic non-fractured trabecular bones.
(XLSX)

S7 Table Statistical analyses of Table 4. P-values of the microscale BMD and microstructure characterization in osteoporotic non-fractured trabecular bones.
(XLSX)

S8 Table Statistical analyses of Table 5. P-values of the chemical composition characterization in osteoporotic non-fractured trabecular bones.
(XLSX)

S9 File Full ARRIVE 2.0 Guidelines checklist. (PDF)
